# Between Scylla and Charybdis?—Health insurance claims-data to monitor quality of service delivery in ophthalmology

**DOI:** 10.1038/s41433-024-03333-5

**Published:** 2024-09-18

**Authors:** Martin K. Schmid, Dawn A. Sim, Stefan Boes, Thomas J. Wolfensberger, Lucas M. Bachmann, Katja Hatz, Michael A. Thiel

**Affiliations:** 1 Eye Clinic, Lucerne Cantonal Hospital LUKS, Lucerne 16, Switzerland; 2 University of Zurich, Zurich, Switzerland; 3 Department of Health Sciences and Medicine, University of Lucerne, Luzern, Switzerland; 4 Institute of Ophthalmology, University College London, London, UK; 5 Genentech South San Francisco, South San Francisco, CA USA; 6 Jules Gonin Eye Hospital, University of Lausanne, Lausanne, Switzerland; 7 Medignition AG, Zurich, Switzerland; 8 Vista Eye Clinic Binningen, Binningen, Switzerland; 9 Faculty of Medicine, University of Basel, Basel, Switzerland

**Keywords:** Education, Health services

## Abstract

The seminal work of Wennberg and Gittelsohn in 1973 emphasised the importance of health information for informed decision-making. This led to the creation of the Dartmouth Health Atlas in 1996, which has become an important resource for monitoring health services in the USA. The Dartmouth Health Atlas research revealed the existence of variation in health care without benefit to patients, and the dependence of health care use on local resource supply. Similar initiatives emerged around the world, from the UK to Asia. The availability of administrative data has become essential for evaluating health service delivery and for informing health economic analysis and policy decisions. Access to data depends on the organisation of the health system, with more centralised systems facilitating comprehensive data collection. We contrast the decentralised structure of the Swiss healthcare system with that of the US and the UK, and highlight the challenges of harmonising data for nationwide health monitoring. The example of optical coherence tomography (OCT) in Swiss ophthalmology illustrates the variability in care practices and billing patterns. This variability can be attributed to the lack of clear guidelines and the complexity of billing codes. Incentives to charge incorrect rates influence billing, adding a further variance component to the variance in care that cannot be subtracted from the total variance at the level of a health insurance fund and distorting the results. In certain environments the quality of data on care is so variable that a sound conclusions for health policy decisions represent a great challenge.

## The golden age of data-driven decision making

In 1973, in their seminal publication in Science, Wennberg and Gittelsohn highlighted the importance of health information about total populations for sound decision-making and planning [1]. Their work eventually led to the Dartmouth Health Atlas in 1996, which has become a pivotal source to monitor and manage health service delivery in the United States of America [2]. The research led by the Dartmouth Health Atlas team has several key findings: Variation in healthcare delivery exists and has no benefit for patients. If the evidence supporting an intervention is weak, variation in care reflects physicians’ rather than patients’ preferences. The extent of health service utilization depends on the local supply of resources, and more health care might not be better [3]. These findings from the USA have had a strong impact and led to similar initiatives in the United Kingdom, Europe, South America, Asia, and Oceania in the last two decades [3, 4].

### Data availability as a catalyst for research

An important driver for the development of the Dartmouth Health Atlas was the availability of Medicare and Medicaid data, providing a comprehensive coverage of health service delivery data for the elderly and the low-income population in the United States [3, 5]. To date, administrative data have become an indispensable resource to assess service delivery in healthcare. In the literature the terms “health care utilization data”, “billing records”, “administrative claims data”, or simply “claims data” are often used interchangeably to describe all sorts of administrative data and billing purposes derived from the health care sector [6].

Based on Wennberg’s ground-breaking work on analysing the variability of care and its impact on healthcare, the use of administrative data to measure the quality of care has taken off. Hardly any health economic analysis can do without this data (summarised in [7]). Political decisions on the organisation of the healthcare system are based on this data. Recently, there have been calls for the assessment of the effectiveness of drugs to be based not only on the results of controlled studies, but also on the effects observed in routine clinical practice [8].

## Data access and potential

Access to administrative data depends heavily on the organisation of the healthcare system (summarised in [9]). The more centralised the control system is, the more likely it is that care data can be collected uniformly and comprehensively. In addition to the conditions in the USA, where the introduction of Medicare and Medicaid in 1965 and the availability of Surveillance, Epidemiology, and End Results (SEER) [5] created important circumstances for the work of Wennberg and colleagues and many other initiatives including cancer surveillance [10], the Danish healthcare system and the British healthcare system following the introduction of the National Health System NHS in 1948 should be mentioned in Europe [11]. In Denmark, the establishment of an agency that oversaw the agreement between industry partners and service providers on the use of a uniform software standard in 1994 led to the development of the Danish Quality Programme (summarised in [12]).

From a methodological point of view, the use of administrative care data is associated with challenges. The harmonisation and standardisation of data collection and formats is an important factor for the validity of the statements [9]. It is therefore no coincidence that healthcare systems that have adopted a top-down approach to planning and monitoring healthcare have been able to implement quality programmes more easily.

The collection of real-world data is also playing an increasingly important role in assessing the efficacy and safety of new therapies. It has long been recognised that the results of controlled clinical trials, which play a crucial role in the approval of a new medicine, can only partially reflect the benefits in routine clinical practice. The collection of real-world data has become increasingly important, not only for post-marketing surveillance and pharmacovigilance, but also for efficient trial design, drug labelling, and the approval of new therapies. Recently, even regulatory authorities have begun to require it prior to approval [13].

## Organisation of healthcare or form follows function

The Swiss healthcare system enjoys an excellent reputation by international standards [14]. In contrast to the USA or the UK, the system has a federal structure. The planning and management of the healthcare system is highly decentralised and regulated at cantonal level in important respects [15]. As a result, 26 different healthcare systems with different regulations provide healthcare in Switzerland, a relatively small country with a population of around 9 million. The federal structure has the great advantage that the local needs of the population can be better considered. However, the implementation of national healthcare programmes poses a challenge [16]. In many cases, it is the different regulations in the cantons or the different processes that hinder nationwide health monitoring. This also applies to national surveys of the quality of care. In Switzerland and other countries, attempts to implement the Wennberg and Dartmouth Health Atlas approaches have been made. In Switzerland, such an initiative has been recently re-launched, but still faces several problems [17].

In contrast to the prevailing care structures, Swiss health insurance funds insure patients across all cantons [15]. This makes them an important player in the healthcare system, with not only a cantonal but also a national view of care. The large health insurance funds in particular have become increasingly involved in the health policy debate and established quality programmes in the wake of the cost trend in the Swiss healthcare system. Billing data is used in co-operation with the health insurance funds for many questions of health services research [18–22]. Research with this data has the advantage that statements can be made across the cantons. However, as the health insurance funds do not have access to the clinical data of their policyholders, the possibilities for analysing the causes of cost variability between service providers and different care structures are limited [19, 20]. Research-based joint ventures between health insurance funds and service providers, in which the cost data is linked to the clinical data of the insured persons of this service provider, are currently the only way to carry out further investigations into cost variability. However, these analyses are complex and must be well justified for data protection reasons [21, 22].

In the United Kingdom, the introduction of a retinal thickness threshold of >400 microns for the treatment of diabetic macular oedema with anti-VEGF drugs, based on a medico-economic analysis within a NICE guideline [23], has led to a vigorous debate about the optimal setting of treatment standards based on controlled clinical trials [24–26]. The analysis of real-world data demonstrated that this treatment threshold and delayed treatment with anti-VEGF drugs resulted in suboptimal visual outcomes for patients [25, 26].

In the discussion about the remuneration of medical services in Switzerland, health insurance billing data is often used. They are used to set benchmarks and to better understand and discuss the variability in care. Based on the work of Wennberg and colleagues, the aim is to identify and analyse conspicuous billing behaviour in the discussion with service providers. When analysing the use and billing of optical coherence tomography (OCT) in patients with retinal diseases in Switzerland, an interesting situation arose pointing to an additional, presumed driver for variability in the claim data.

## Where’s the beef?—The example of optical coherence tomography

In ophthalmology, the OCT is a diagnostic procedure typically used to manage patients with cases of neovascular age-related macular degeneration (nAMD) and diabetic macular oedema (DMO) [27, 28]. All relevant guidelines suggest basing treatment decisions on the results of the OCT scan. While the use of OCT examinations for therapy monitoring is undisputed, the evidence for binocular examinations when only one eye is initially affected by the retinal disease is unclear. There are no binding recommendations from guidelines or professional associations [27, 28]. The billing of OCT services is handled differently internationally. OCT examinations are often billed as a lump sum. This means that it is not clear from the billing data whether one or both eyes were examined. In Switzerland, the OCT examination is billed separately for each eye, which makes it possible to analyse how it is handled. The analysis of the billing data from the health insurance companies showed a high degree of variability between the individual service providers and billing patterns that were difficult to interpret. This prompted us to conduct an anonymous survey of the largest providers of retinal disease services in Switzerland to find out how they arrange OCT examinations for monocular nAMD or DME and how these services are billed. The responses from the 15 largest institutions, which together provide around two thirds of care, revealed interesting behavioural patterns.

### Variability in care

We found that four out of five centres performed bilateral OCT at least every three months for both nAMD and DME patients. Half of the centres reported performing bilateral OCT scans at every visit for DME and two-thirds for nAMD. One-fifth of centres reported performing bilateral OCT scans only when there were clinical signs or symptoms, such as deterioration in vision or fundoscopic findings suggesting the onset of disease in the untreated eye.

### Billing patterns

Surprisingly, billing patterns did not match clinical practice and varied widely. Only one centre billed each bilateral OCT with the correct code for a bilateral examination. Four centres reported that they examined the untreated eye free of charge, and other centres billed for OCT in the untreated eye only periodically (once or twice a year). In addition, one third of the centres for nAMD and almost half of the centres for DME treated OCT in the untreated eye as a contingency and only billed for it if the OCT showed progression of the disease. Finally, three centres never billed for OCT for nAMD and one centre never billed for DME. Instead, they used the cheaper billing code for retinal photography. Billing patterns for treated eyes were consistent across centres. However, a quarter of centres did not always use the correct code for OCT in the anti-VEGF treated eye, sometimes preferring to use the incorrect, but significantly cheaper, billing code for retinal photography.

## Is good not good enough?

Half a century of health systems research has contributed significantly to our understanding of health systems and health care [29]. For research with administrative data, such as health data, to work well, certain framework conditions must be met. Switzerland is an interesting example. As already known from other studies, this survey also shows variability in healthcare practice [30, 31]. For our case study, we can only speculate about the causes of this variability. The international literature on the correct use of OCT examinations in the second eye is controversial. In addition, there is a lack of binding guidelines that could serve as a guide for care providers [27, 28]. If guidelines only provide vague recommendations, this is likely to lead to increased variability. Specialist societies can also make an important contribution to improving data quality. For example, the Swiss Vitreoretinal Group has not yet commented on how the approximately 300 ophthalmologists in Switzerland who offer intravitreal injections should proceed in this situation. The tariff system on which the health claim data is based must be unambiguous and provide clear guidelines for billing [32]. Service providers must undertake to adhere to these guidelines. If, for example, disincentives to charge the wrong rates influence billing, a further variance component is added to the variance of care provision, which cannot be subtracted from the total variance at the level of a health insurance fund and distorts the results [32]. For a well-founded analysis of the variability of care, it is also important to have information on the initial clinical situation to examine the heterogeneity in the data [7].

The 21st century is also the digital century in medicine. We will have more and more opportunities to make data-based statements about the quality of healthcare easier and faster. This vision is often still associated with fears, particularly among service providers. Clear rules and objectives for care analysis are important prerequisites for reducing these fears. The continuous analysis of the quality of care is an important building block for ensuring the long-term health of the population and providing patients with optimal care.
